# The Association Between the Onset and Ending of Volunteering on Loneliness and Perceived Social Isolation Among Older Adults: Longitudinal Evidence From the German Ageing Survey

**DOI:** 10.1002/brb3.70244

**Published:** 2025-01-09

**Authors:** Avery Richardson, Hans‐Helmut König, André Hajek

**Affiliations:** ^1^ Department of Health Economics and Health Services Research University Medical Center Hamburg‐Eppendorf, Hamburg Center for Health Economics Hamburg Germany

**Keywords:** loneliness, older adults, social isolation, voluntary work, volunteering

## Abstract

**Background:**

Existing literature explores the relationship between voluntary work, loneliness, and social isolation, but there is a lack of research on how the onset and cessation of voluntary work relate to loneliness and social isolation among older adults. Many in this population may discontinue volunteering due to various life circumstances, making it important to investigate the longitudinal significance of these transitions. This study aims to assess whether engaging in volunteer work during retirement age is associated with changes in loneliness and social isolation.

**Methods:**

Longitudinal data were obtained from Waves 5 (Year 2014) and 6 (Year 2017) of the German Ageing Survey, focusing on middle‐aged and older adults. The sample size (*n* = 6628) was limited to those aged 65 and above. Two groups were analyzed: the onset group, individuals who did not volunteer in 2014 but did by 2017 (188 individuals), and the cessation group, those who volunteered in 2014 but not by 2017 (307 individuals). Loneliness was assessed using the De Jong Gierveld tool, and perceived social isolation was measured using the Bude and Lantermann instrument. Asymmetric linear fixed effects (FE) regression analysis examined the associations.

**Results:**

In an asymmetric FE regression analysis that adjusted for a multitude of time‐varying covariates, an association was shown between the onset of volunteer work and decreases in loneliness (*β* = −0.07; *p* = 0.04) in older adults. In contrast, there was no significant association between the onset of voluntary work and changes in perceived social isolation. Also, there was no significant association between the cessation of volunteer work and changes in perceived social isolation or loneliness.

**Conclusion:**

Our findings suggest that older adults who choose to volunteer may experience a decrease in self‐reported loneliness. Further longitudinal studies are needed to confirm our present findings.

## Graphical abstract

This study examined how starting or stopping volunteering is associated with loneliness and social isolation in older adults. Using data from the German Ageing Survey, results showed that those who began volunteering felt less lonely. Findings suggest volunteering may help older adults feel more connected, though future research is needed.

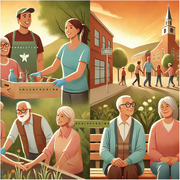

## 1 Introduction

Volunteering is the term used to describe unpaid work performed voluntarily in order to serve the community's best interest (Simonson et al. 2021). There are numerous positions that volunteers can take on with varying time commitments. For example, they can perform community service, resolve disputes between neighbors, educate children, put out fires, feed the hungry, or even organize meetings for religious rites and local events (Simonson et al. 2021). According to information obtained for the Fifth German Survey on Volunteering, approximately 40% of the country's population aged 14 and over, or over 28 million people, reported volunteering in Germany in 2019 (Simonson et al. 2021).

According to the Fifth German Survey on Volunteering, the rate of volunteering among people 65 and older grew the most between 1999 and 2019 compared to other age groups, rising by 13.2 percentage points, from 18.0% in 1999 to 31.2% in 2019. Additionally, the Fifth German Survey on Volunteering also stated that although this age group had the lowest total volunteer rate in 2019, it did have the greatest percentage (at 22.2%) of volunteers who spent 6 or more hours per week on their volunteer activity.

Depending on the circumstances of the volunteer placement, volunteering can improve social, physical, and cognitive functioning to varying degrees (Anderson et al. 2014). A better level of general life satisfaction may result from volunteering, one study showed (Huo and Kim 2022). Additionally, according to the World Health Organization, healthy aging is defined as the preservation of functional abilities that promote well‐being in later life, such as the ability to meet basic needs, learn, develop, and make decisions, be mobile, form and maintain relationships, and contribute positively to society (Akhter‐Khan et al. 2023). Consequently, it may be said that volunteering is essential to being a healthy older adult.

Although various aspects of volunteering have been studied, it is poorly known how volunteering influences feelings of loneliness and perceived social isolation. Based on data from a 2016 multi‐item survey of older adults in the United States of America (*N* = 9944; mean age = 75.94 years, SD = 7.70 years; 59.4% females), Lee's (2022) cross‐sectional research suggested that two types of volunteer work, volunteer youth work and charity work, strongly predicted lower levels of loneliness in older adults. Carr et al. (2018) conducted a study using cross‐sectional data, which included 5882 participants with a mean age of 67.9 years (SD = 8.41 years) and a sample that was 53.2% female. The results indicated that volunteering for 2 or more hours per week was associated with reduced levels of loneliness in recently widowed older adults. These levels of loneliness were comparable to those of individuals who had consistently remained married. In contrast, volunteering for less than 2 h per week or not volunteering at all did not have the same effect. When compared to older persons who volunteered less than 100 h annually or did not volunteer at all, Cho and Xiang (2022) discovered that those who engaged in moderate or high intensity volunteering (defined as 100 h or more annually) were considerably less likely to feel lonely. Findings were based on longitudinal data from the general older adult population throughout the United States (*N* = 5000; mean age = 74.7 years, SD = 0.19; 56.4% females), which started in 2006.

Regarding significance, perceived social isolation and loneliness have both been linked to cardiovascular disease, hypertension, obesity, depression, early mortality, cognitive decline, and Alzheimer's disease (Coyle and Dugan 2012; Huo and Kim 2022). In actuality, the mortality risk associated with a lack of social connections is comparable to that associated with smoking, almost twice as much as that associated with obesity, and four times as much as that associated with exposure to air pollution (Holt‐Lunstad, Smith, and Layton 2010). Volunteering can help to reduce the risk of loneliness, which can result in serious health issues (Cacioppo and Cacioppo 2014).

When a person's desired and actual social connections are at odds, loneliness results (Hawkley and Cacioppo 2010). The depth of loneliness experienced by an individual may vary based on their interpretation of what society deems as a healthy level of social participation (Tesch‐Roemer and Huxhold 2019). This suggests that one person may perceive their level of loneliness differently from another, depending on their personal understanding and expectations regarding social engagement. For instance, some people prefer a small number of close friends to feel connected, while others prefer large social networks. Therefore, perceived deficiencies in a relationship's quality, its usefulness (i.e., perceived social support), and the size of one's social network may all contribute to loneliness (Boger and Huxhold 2018a, 2018b).

Those who do not feel like they belong in society may experience perceived social isolation, which commonly keeps them from participating in social activities that society values (MacLeod et al. 2019). Those who perceive themselves as unfit to be part of society as a whole may often have a gloomy outlook on their chances for future social participation, which could lead to increasing degrees of perceived social isolation (Hommerich 2015).

Social isolation and loneliness, while closely related, differ in their sociocultural structures and theoretical foundations. Perceived social isolation is influenced by societal factors, whereas loneliness focuses on individual interpersonal dynamics (Huxhold, Suanet, and Wetzel 2022). Despite these differences, both concepts involve feeling disconnected or lacking belonging. Due to their interconnectedness, these concepts may also gradually reinforce one another. For instance, experiencing a sense of disconnection from one's personal network of social connections can contribute to a diminished feeling of belonging within society as a whole. Conversely, feeling detached from the broader community may intensify feelings of social isolation within personal relationships, leading to an increased sense of loneliness (Huxhold, Suanet, and Wetzel 2022). This underscores the interconnectedness between personal relationships and broader societal belonging while acknowledging the unique sociocultural structural levels and theoretical underpinning principles of both loneliness and social isolation.

Examining both loneliness and social isolation is crucial when assessing the association with volunteering for several reasons. First, loneliness and social isolation are closely related but distinct concepts that can have overlapping yet also unique consequences on an individual's well‐being and quality of life. While loneliness primarily revolves around subjective feelings of disconnection and lack of social support, objective social isolation refers to objective aspects of social connectedness, such as the size of social networks (and perceived social isolation refers to a feeling of not belonging to the society). Therefore, understanding both dimensions allows for a comprehensive understanding of how volunteering may influence individuals’ social experiences and perceptions.

Furthermore, volunteering activities can potentially shape loneliness and social isolation through various mechanisms (Moen, Robison, and Dempster‐McClain 1995; Rozario, Morrow‐Howell, and Hinterlong 2004). For example, volunteering may provide opportunities for social interaction and the development of meaningful relationships, which can mitigate feelings of loneliness. Additionally, volunteering can foster a sense of belonging and social integration within communities, thereby reducing social isolation (Luque‐Suárez et al. 2021). By incorporating both loneliness and social isolation into our study, we explored the multifaceted ways in which volunteering may contribute to an individual's social well‐being (Huang 2019; Matthews and Nazroo 2021; Richardson, König, and Hajek 2023).

Previous research on the topic of volunteering and loneliness primarily consisted of cross‐sectional studies (i.e., looking at a certain point in time) conducted in the USA or Asia, leaving a gap in understanding longitudinal dynamics. Therefore, our study aimed to address this gap by utilizing longitudinal data, allowing us to track developments and shifts in the characteristics of the target group over time. To the best of our knowledge, there is an absence of studies that examine the association between the onset and the cessation of voluntary work, loneliness, and perceived social isolation, particularly in Europe. The objective of this study was to examine whether volunteering in retirement age (65 years and above) is associated with a decrease in levels of loneliness and perceived social isolation.

Given the likelihood of individuals discontinuing volunteering due to various life circumstances, it is crucial to examine the potential longitudinal significance of these transitions. To achieve our objective, we identified two groups for our analysis: the onset group—those who did not report volunteering in 2014 but started doing so by 2017, and the cessation group—individuals who reported volunteering in 2014 but stopped by 2017.

Understanding the unique aspects of the onset and cessation of volunteering is crucial for comprehensively assessing their association with individual's sense of loneliness and social isolation. The decision to start or stop volunteering can be influenced by a myriad of factors, including personal motivations, health considerations, changes in social networks, and life transitions such as retirement. The onset of volunteering represents a proactive engagement with the community, often driven by a desire to contribute, establish social connections, or find purpose in retirement. On the other hand, the cessation of volunteering may stem from various reasons, such as declining health, time constraints due to newfound caregiving responsibilities, relocation, or shifts in priorities. Additionally, the experience of transitioning out of volunteer roles can vary widely among individuals, with some experiencing a sense of loss or disconnection from their social networks, while others may remain confident to find alternative ways to stay engaged. Examining the onset and cessation of volunteering allows for a nuanced understanding of how these transitions are associated with individuals’ social connections, sense of purpose, and experiences of loneliness and social isolation over time.

## 2 Methods

### 2.1 Sample

This study employed information from the nationally representative German Ageing Survey (DEAS, also known as the “Deutscher Alterssurvey”). The German Centre for Gerontology (DZA) (n.d.) “Deutsches Zentrum für Altersfragen” has been administering the poll since 2002 with funding from the Federal Ministry for Family Affairs, Older Citizens, Women, and Youth (BMFSFJ). By combining longitudinal follow‐ups every 3 years with subsequent representative sampling every 6 years, the DEAS employs a cohort‐sequential technique.

The DZA (n.d.) stated that although the DEAS has a number of goals, it focuses particularly on the relationship between diversity, social injustice, and the quality of later life. The primary objective of the DEAS is to evaluate how aging impacts people in Germany. The fundamental strategy for achieving this is to look at how society and people change throughout time (DZA n.d.). In 1996, adults living in a community who were 40 years of age or older were initially chosen from registrations of the general population. Participants from the initial wave of the survey were invited to participate in seven additional waves, which were conducted in 2002, 2008, 2011, 2014, 2017, 2020, and 2021. Baseline samples were collected in Waves 2 (in 2002), 3 (in 2008), and 5 (in 2014) of the cohort‐sequential design of the DEAS project. Comparatively, Waves 4 (2011) and 6 (2017) were panel surveys. Aside from general health, well‐being, and life satisfaction, the surveys also inquire about respondents’ employment and retirement status, income, assets, and inheritances, ability to deal with digitalization and technology, social networks, including family, partnerships, and relationships, informal help, support, and care from family members, as well as their attitudes and views on aging and ageism stereotypes (DZA n.d.). Information from a computer‐assisted personal interview (CAPI), which is conducted by trained interviewers at the participant's home, is utilized in the survey. Additionally, it includes use of information from a questionnaire that participants are provided to complete on their own following the interview (with the option of completing it online). The study gathers data via the CAPI on socioeconomic and demographic characteristics, living arrangements, and other aging‐related topics. Data on subjective opinions, psychological themes, and health‐related information are collected through the participant‐completed questionnaire. The response rate for all panel samples (from the years 1996, 2002, 2008, and 2014) was 63%.

In this current study, we used data from Waves 5 and 6. We restricted our sample to those in retirement age (65 years and above); thus, we were left with 6628 in the analytical sample. Surveys were distributed to all participants between April and November of the respective year. Each participant gave their written, informed consent. An ethics vote is not necessary for this investigation as the criteria for such a vote are not met (e.g., use of invasive methods). We classified the sample into the following two groups for our analysis: the onset group, which consisted of people who did not report volunteering in 2014 but did so by 2017, and the end group, which consisted of people who did report volunteering in 2014 but did not do so by 2017. The onset group consisted of 188 individuals and the end group consisted of 307 individuals.

### 2.2 Measures

Loneliness was assessed using the six‐item De Jong Gierveld Loneliness Scale (De Jong Gierveld and Van Tilburg 2006). Each item encompassed four response categories, ranging from “strongly disagree” to “strongly agree.” The scale contained three negatively formulated items (“I experience a general sense of emptiness,” “I miss having people around,” and “Often, I feel rejected”) and three positively formulated items (“There are plenty of people that I can lean on in case of trouble,” “There are many people that I can count on completely,” and “There are enough people that I feel close to”) (De Jong Gierveld and Van Tilburg 2010). Three items were recoded. Thereafter, a final score was computed by calculating the average of each item. The scores ranged from 1 to 4, with higher values signifying greater levels of loneliness. Cronbach's alpha for Wave 5 was calculated at 0.82, whereas Cronbach's alpha for Wave 6 was calculated at 0.83. This instrument is extensively used and has good psychometric characteristics (De Jong Gierveld and Van Tilburg 2006, 2010).

Social isolation was assessed using the four‐item Bude and Lantermann tool (Bude and Lantermann 2006). The scale consisted of four negatively formulated items (“I feel excluded from society,” “I am worried to be left behind,” “I feel that I am left out,” and “I feel like I do not really belong to society”) (Hajek and König 2019a). Respondents were asked to rate each item from 1 *(strongly agree*) to 4 (*strongly disagree*) based upon their agreement level with each statement. Each item was recoded. A final score was generated by calculating an average score from the sum of each item's scores. The scores ranged from 1 to 4, with higher values signifying greater levels of social isolation.

### 2.3 Independent Variables

The presence of volunteer work was measured using a binary scale (1 = *yes* and 0 = *no*). Participants were asked during an oral interview whether they formally or informally volunteer for a responsibility in any of the clubs or organizations to which they belong. Occupational interest groups, volunteer rescue services, volunteer fire departments, healthcare organizations, and political interest groups are a few examples of formal volunteering (voluntary work done with a group or organization). Examples of informal volunteering (volunteer labor that is carried out without the assistance of a group or organization) include serving as a lay judge, a parent spokesman, or a representative for a local charity group. Amount of voluntary work hours was not utilized in this study due to data limitations.

### 2.4 Covariates

In ways that are consistent with previous studies (Carr et al. 2018; Lee 2022; Cacioppo and Cacioppo 2014), the following time‐varying factors were controlled for in the regression analysis of this study:

Age (in years), marital status (married, living with spouse; married, living apart from spouse; single; divorced; widowed), employment status (employed; retired; other), and household net equivalent income (log income was calculated) were all taken into consideration when including time‐varying socioeconomic factors. Smoking and alcohol consumption were considered to be time‐varying lifestyle‐related factors. Smoking was quantified using a subscale of the German National Health Survey (Bellach 1999). Alcohol was quantified using a qualitative scale, ranging from 1 (*daily*) to 6 (*never*). Depressive symptoms, self‐rated health, functional health, and the number of chronic conditions were all considered time‐varying health‐related criteria. The Center for Epidemiologic Studies Depression Scale (CES‐D, 15‐item version) was used to measure depressive symptoms (Van Dam and Earleywine 2011). Higher values of the sum score, which ranges from 0 to 45, indicate more depressed symptoms. A single‐item measure was used to quantify self‐rated health where individuals were asked to rate their current health on a 5‐point Likert scale from 1 (*very good*) to 5 (*very bad*; Hajek and König 2019b). A count score was used to quantify chronic conditions (ranging from 0 to 11, with higher values corresponding to more chronic conditions). Included on the list of chronic conditions on the drop‐off survey were cardiac and circulatory disorders [0/1]; bad circulation [0/1]; joint, bone, spinal, or back problems [0/1]; respiratory problems, asthma, and shortness of breath [0/1]; stomach and intestinal problems [0/1]; cancer [0/1]; diabetes [0/1]; gall bladder, liver, or kidney problems [0/1]; bladder problems [0/1]; eye problems and vision impairment [0/1]; and ear problems and hearing problems [0/1]. Measures of functional health were based on the physical functioning subscale of the SF‐36 health survey questionnaire (Short Form Health, 36‐item version; Brazier et al. 1992), a self‐administered questionnaire with 36 items spread across eight distinct domains. For each dimension, item scores are coded, tallied up, and then converted to a scale from 0 (*lowest health*) to 100 (*best health*).

For descriptive purposes, the time‐constant factors of sex (men and women) and education (ISCED‐97) (Organisation for Economic Co‐Operation and Development 1999) classification distinguishing between low, medium, and high education were used. In contrast to time‐varying factors, time‐constant factors are factors that do not vary within individuals over time.

### 2.5 Statistical Analysis

Sample characteristics for the analytical sample are presented in total, pooled over the waves used. Subsequently, asymmetric linear regressions with fixed effects (FE) were performed to explore the association between the onset and cessation of voluntary work and loneliness, as well as perceived social isolation, while adjusting for several time‐varying covariates.

Compared to other panel regression models, such as random effects regressions, FE offers several advantages. Even in the presence of time‐constant factors, both observed and unobserved, FE regressions yield consistent estimates (Brüderl and Ludwig 2014). Notably, FE regressions exclusively analyze intraindividual changes over time, such as from Waves 5 to 6, (capturing shifts in volunteer activity within individuals). Thus, since the focus of FE estimates is on intraindividual changes over time, it refers to an average treatment of the treated. This focus on such changes is not a shortcoming of the FE approach; rather, it reflects the fact that only a subset has such changes over time in the population. It is important to emphasize that only individuals who participated in both Waves 5 and 6 (and had changes within this period) contribute to the beta‐coefficients in our FE regression model.

Traditionally, FE models assume symmetric effects of variables. For example, this would imply that the result of the onset of volunteering on perceived social isolation and loneliness is the same as the result of the cessation of volunteering (in absolute terms). As asymmetry may be more credible (Allison 2019), our study employs asymmetric FE regressions to discern between the onset and cessation of volunteering, particularly focusing on volunteering transitions. The significance level was set at *p* < 0.05. Stata 17.0 (Stata Corp., College Station, TX) was used for statistical analyses.

In light of the methodological considerations, it is important to further elucidate our rationale for employing asymmetric FE regressions in our analysis. While we acknowledge that alternative statistical models might have been applicable, we contend that the use of asymmetric regression with FE is justified for several reasons. First, asymmetric regression allows us to account for time‐invariant individual characteristics, which is essential for controlling for time‐constant unobserved heterogeneity that may bias our estimates. Moreover, the use of asymmetric regression with FE is well‐suited for analyzing continuous outcomes, even if they are measured on a 4‐point scale. For instance, while ordered logit models are typically employed for ordinal outcomes with ordered categories, our decision to treat the outcomes as continuous variables is justified by the nature of the data and the research question at hand. By treating loneliness and social isolation as continuous variables, we are able to capture the full range of variation in these constructs. Furthermore, asymmetric FE regressions provide several advantages, including ease of interpretation. By using this approach, we are able to directly quantify the longitudinal association between volunteering and loneliness/social isolation while controlling for individual‐specific characteristics and time‐varying factors.

## 3 Results

### 3.1 Sample Characteristics and Bivariate Analysis

Sample characteristics of the analytical sample are shown in Table 1 (*n* = 6628 observations). In the total sample, the average age was 74.0 (range: 65–97; SD: 6.1 years) and 46% were female. Furthermore, average loneliness score was 1.7 (SD: 0.5). Average social isolation score was 1.6 (SD: 0.6).

**TABLE 1:** Characteristics of total analytical sample (pooled over both waves).

Variables	Mean (SD)/*n* (%)
*N* (%)	6628 (100.0)
Sex: *N* (%)	
Men	3580 (54.0)
Women	3048 (46.0)
Age: mean (SD)	74.0 (6.1)
Marital status: *N* (%)	
Married, living together with spouse	4541 (68.5)
Married, living separated from spouse	68 (1.0)
Divorced	547 (8.3)
Widowed	1228 (18.5)
Single	244 (3.7)
Education level: *N* (%)	
Low (ISCED 0–2)	493 (7.4)
Medium (ISCED 3–4)	3278 (49.5)
High (ISCED 5–6)	2856 (43.1)
Employment status: *N* (%)	
Working	60 (0.9)
Retired	6390 (96.4)
Other: not employed	178 (2.7)
Monthly household net income: mean (SD)	2553 (18.6)
Smoking: *N* (%)	
I smoke daily	456 (6.9)
I smoke occasionally	167 (2.5)
I used to smoke, but not anymore	2693 (40.6)
I have never smoked	3312 (50.0)
Alcohol intake: *N* (%)	
Daily	965 (14.6)
Several times a week	1525 (23.0)
Once a week	892 (13.5)
1–3 times per month	741 (11.2)
Less often	1657 (25.0)
Never	848 (12.8)
Number of chronic conditions: mean (SD)	3.1 (2.0)
Self‐rated health: mean (SD)	2.6 (0.8)
Functional health: mean (SD)	77.3 (24.7)
Depressive symptoms: mean (SD)	6.4 (5.6)
Loneliness: mean (SD)	1.7 (0.5)
Perceived social isolation: mean (SD)	1.6 (0.6)

*Note*: Sex and education were omitted from fixed effects regressions because they are time constant. Thus, they remain constant throughout time for each individual.

**FIGURE 1:**
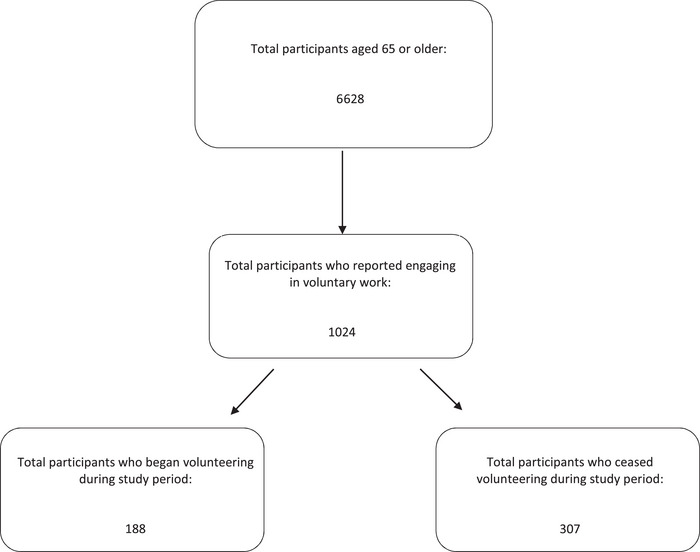
Composition of the sample.

Out of the 6628 participants aged 65 years or older included in the study, 1024 reported engaging in voluntary work at any point during the study period (see Figure 1). Among these participants, 188 individuals began volunteering, while 307 ceased their voluntary engagement. The characteristics of such individuals are displayed in Tables S1 and S2.

### 3.2 Regression Analysis

In Table 2, results of asymmetric FE regressions are shown for the total sample. After adjusting for several time‐varying covariates, FE regression analyses revealed a negative association between the onset of voluntary work and loneliness (*β* = −0.07; *p* < 0.05) in older adults. However, FE regression analyses did not reveal a significant association between the onset of voluntary work and social isolation (*β* = −0.03). Similarly, FE regressions showed that neither loneliness (*β* = 0.00) nor social isolation (*β* = −0.02) were associated with the end of volunteering over time.

**TABLE 2:** The onset and end of voluntary work, loneliness, and social isolation in older adults. Results of asymmetric linear FE regressions.

	Loneliness—total sample	Social isolation—total sample
Onset of voluntary work	−0.07^*^ (0.03; −0.01 to −0.13)	−0.03 (0.05; −0.13 to 0.07)
Cessation of voluntary work	−0.00 (0.03; −0.06 to 0.06)	0.02 (0.04; −0.06 to 0.10)
Covariates	✓	✓
Constant	1.51^***^ (0.38)	0.97^*^ (0.45)
*R* ^2^	0.02	0.02
Observations	6628	6613
Number of individuals	4592	4576

*Note*: Unstandardized beta coefficients are reported; robust standard errors and 95% CI are given in parentheses; and covariates (✓) include age, marital status, employment status, smoking status, alcohol intake, presence of chronic illnesses, self‐rated health, functional health, and depressive symptoms.

With regard to the number of individuals (which is not equal to the number of observations divided by 2), it is important to highlight that the Stata command used for linear fixed effects (FE) regressions (achieved through “xtreg” using the “fe” option) incorporates units (specifically, individuals) with only one observation when calculating the total number of observations. This inclusion of single‐observation units is significant because these units contribute information about various metrics such as the between‐group *R* ^2^, overall *R* ^2^, variance components, constant term, and the relationship between individual effects that remain constant over time and the explanatory variables. However, it is also worth underlining that this inclusion does not impact the beta coefficients and their corresponding standard errors.

* *p* < 0.05.

** *p* < 0.01.

*** *p* < 0.001.

+*p* < 0.10.

In a sensitivity analysis, an FE‐ordered logit model for panel data (i.e., the “blow‐up and cluster” estimator from Baetschmann, Staub, and Winkelmann 2015) was used instead of the linear FE model (Baetschmann et al. 2020). This model still showed a significant association between the onset of voluntary work and loneliness (*p* = 0.02). All other associations of interest remained insignificant.

## 4 Discussion

This study sought to evaluate the association between the onset and end of volunteering, loneliness, and social isolation in older adults based on longitudinal data from a large, nationally representative sample of adults in Germany. An association between the onset of volunteer activity and decreases in loneliness was identified via asymmetric linear FE regressions. The onset of volunteering, however, was not associated with a significant change in social isolation nor was the end of volunteering associated with a significant change in loneliness or social isolation. This study markedly advanced our current understanding by investigating the association between the onset and end of voluntary engagement, loneliness, and social isolation among older adults in Germany. The majority of studies on this topic have used sample data from cross‐sectional studies based in the USA or Asia. The findings of this study are in accordance with those of prior studies that found that voluntary engagement may have the potential to decrease loneliness (Akhter‐Khan et al. 2023; Carr et al. 2018; Lee 2022). This is crucial because it implies that encouraging older people to volunteer may help to reduce levels of loneliness.

Beginning a volunteer program is associated with decreases in loneliness among persons over 65. It is possible that volunteering has positive mid‐term implications on social skills that are maintained even after volunteering stops, which may allow these individuals to go back to their former level of loneliness. These observations might be justified by set point theories (Lucas et al. 2004). According to set point theories of subjective well‐being, after responding to events, people eventually return to their baseline levels of happiness and satisfaction (Lucas et al. 2004). Meaning that after stressful (and favorable) life experiences, humans frequently return to their established levels of happiness (Lucas et al. 2004). Retirement and coping with grief and loss are two important lifestyle changes that frequently influence this age group. It is possible that volunteering may equip older adults with skills needed to return to their baseline levels of loneliness after experiencing a life‐changing event. Additionally, volunteering is likely to help people enhance their social skills, which might then increase their self‐confidence in social situations (Lee 2022). This influence can lead individuals to look for additional social engagement opportunities in addition to their current voluntary work. As a consequence, one's social network may grow and they may become more socially integrated, which will increase their self‐efficacy for socializing (Burger and Teets 2004; Casiday, Kinsman, and Fisher 2008). These experiences may eventually result in a reduction in loneliness. This association thus may be explained by variables such as the size of one's social networks and the frequency of one's social interactions (Messias, De Jong, and McLoughlin 2005).

Interestingly, we observed a statistically significant negative association between the onset of voluntary work and loneliness in older adults (*β* = −0.07; *p* < 0.05). While this finding is statistically significant, the practical significance of the observed change may be modest. However, it is crucial to recognize that even small changes in loneliness can hold considerable meaning, particularly among older adults who may be more susceptible to loneliness and its associated health consequences (Malcolm, Frost, and Cowie 2019; Pengpid and Peltzer 2021). Our findings may carry important implications for the well‐being of this population. Moreover, it is essential to consider the cumulative consequences of volunteering over time and its potential role in fostering social connections and reducing loneliness among older adults (Warner et al. 2023). Further research investigating this cumulative significance is warranted.

Ceasing voluntary engagement was not significantly associated with changes in perceived social isolation or loneliness in older adults in our study, thus, this highlights the potential importance of beginning volunteering. This age group is likely to experience several life‐altering events that could alter one's capacity for and desire to maintain volunteer commitments (e.g., loss of a spouse, debilitating physical injury, sudden decline in health status, and unexpected financial strain). Additionally, it is possible that individuals in this age group may not regard volunteering as particularly important in terms of their social lives. In this age group, family caregiving may prove to be more beneficial at preventing loneliness than continuing to volunteer (i.e., providing grandchild care and spousal caregiving: assisting with physical recovery or household tasks) (Burger and Teets 2004; Organisation for Economic Co‐Operation and Development 1999).

Some of the following strengths and limitations are worth noting. The DEAS's main advantages are the sizable and representative samples of Germans who live in communities and are at least 40 years old (Klaus et al. 2017). This current study is unique in that it is the first to analyze the beginning and end of volunteer activity using longitudinal data from DEAS and two valid outcome measures (social isolation and loneliness). Moreover, the covariates included in this study were chosen after considering theoretical considerations and prior studies.

There are a few drawbacks with the DEAS. The interview is conducted exclusively in German, the majority of the data is based on self‐reported information, and the response rate was relatively low. Nonetheless, as mentioned above, the selection bias is very small and, therefore, DEAS can be regarded as representative for people in Germany who live in communities and are 40 years of age or older (Klaus et al. 2017). Future research may also be interested in the quantity of volunteer hours contributed, the position held within a volunteer group, and the duration of volunteer membership. Moreover, we restricted our studies to loneliness and perceived social isolation. Upcoming studies could also explore a more objective view of social isolation (Courtin and Knapp 2017).

One key limitation of this study is its observational nature, which precludes the assumption or claim of causation. Additionally, FE regressions do not address time‐varying unobservables, such as additional volunteering that may have occurred during the study period. This limitation could potentially influence the estimated beta coefficients. Furthermore, there may have been other changes in socialization habits during the gap in observation periods that were not captured in the analysis. Further research could explore the differentiation between informal and formal volunteering activities, as individuals might have engaged in activities beyond the scope of this study, potentially contributing to the observed decrease in loneliness during the gap years (2014–2017).

## 5 Conclusion

The findings of our study offer compelling evidence of a longitudinal negative association between the onset of volunteering and levels of loneliness among older adults. Upcoming longitudinal studies are needed to confirm our present findings.

Understanding the mechanisms underlying this association is important. Further research is needed to explore the specific pathways through which volunteering is associated with loneliness over time, including the role of social support, sense of belonging, and opportunities for social interaction. Additionally, future research may also consider factors such as the quantity of volunteer hours contributed, the position held within a volunteer group, and the duration of volunteer membership.

Moreover, it is important to consider the broader context of social disparities in aging. Underserved populations, including those with low educational attainment and low socioeconomic status, may face unique challenges related to loneliness and social isolation. Studies focusing on these populations can provide valuable insights into how volunteering interventions can be tailored to meet the needs of diverse communities and promote social inclusion and well‐being for all older adults.

In summary, while our study highlights the longitudinal association between volunteering and loneliness among older adults, there is still much to learn about how best to harness these benefits and address the complex issues of loneliness and social isolation in aging populations.

## Author Contributions

**Avery Richardson**: visualization, writing–original draft, writing–review and editing, investigation, conceptualization, methodology, formal analysis. **Hans‐Helmut König**: supervision, project administration. **André Hajek**: conceptualization, project administration, supervision, methodology, formal analysis, writing–review and editing.

## Conflicts of Interest

The authors declare no conflicts of interest.

### Peer Review

The peer review history for this article is available at https://publons.com/publon/10.1002/brb3.70244.

## Supporting information




**Table S1** Baseline characteristics of individuals beginning to volunteer.
**Table S2** Baseline characteristics of individuals ceasing to volunteer.

## Data Availability

Data are subject to third party restrictions. Interested individuals may contact the DZA: https://www.dza.de/en/research/fdz/access‐to‐data/application.
