# Supplementary material for: The Association Between the Onset and Ending of Volunteering on Loneliness and Perceived Social Isolation Among Older Adults: Longitudinal Evidence From the German Ageing Survey
Source: Brain Behav. 2025 Jan 9;15(1):e70244. doi: 10.1002/brb3.70244 (PMC11726694; doi:10.1002/brb3.70244)
Supplement: Supplementary file 1 — Table S1 Baseline characteristics of individuals beginning to volunteer. Table S2 Baseline characteristics of individuals ceasing to volunteer. [file BRB3-15-e70244-s001.docx]

Supplementary Table 1. Baseline characteristics of individuals beginning to volunteer.

| Variables |  | Mean (SD)/n (%) |
| --- | --- | --- |
| Sex: N (%) |  |  |
| Men |  | 103 (54.8%) |
| Women |  | 85 (45.2%) |
|  |  |  |
| Age: Mean (SD) |  | 72.4 (5.5) |
|  |  |  |
| Marital Status: N (%) |  |  |
| Married, living together with spouse |  | 123 (65.4%) |
| Married, living separated from spouse |  | 5 (2.7%) |
| Divorced |  | 22 (11.7%) |
| Widowed |  | 30 (16.0%) |
| Single |  | 8 (4.3%) |
|  |  |  |
| Education Level: N (%) |  |  |
| Low (ISCED 0-2) |  | 11 (5.9%) |
| Medium (ISCED 3-4) |  | 91 (48.4%) |
| High (ISCED 5-6) |  | 86 (45.7%) |
|  |  |  |
| Employment Status: N (%) |  |  |
| Working |  | 3 (1.6%) |
| Retired |  | 179 (95.2%) |
| Other: not employed |  | 6 (3.2%) |
| Monthly Household Net Income: Mean (SD) |  | 2924 (3235) |
|  |  |  |
| Smoking: N (%) |  |  |
| I smoke daily |  | 8 (4.9%) |
| I smoke occasionally |  | 6 (3.7%) |
| I used to smoke, nut not anymore |  | 80 (48.8%) |
| I have never smoked |  | 70 (42.7%) |
|  |  |  |
| Alcohol Intake: N (%) |  |  |
| Daily |  | 29 (17.8%) |
| Several times a week |  | 44 (27.0%) |
| Once a week |  | 20 (12.3%) |
| 1 - 3 times per month |  | 18 (11.0%) |
| Less often |  | 32 (19.6%) |
| Never |  | 20 (12.3%) |
|  |  |  |
| Number of Chronic Conditions: Mean (SD) |  | 2.8 (1.8) |
|  |  |  |
| Self-rated Health: Mean (SD) |  | 2.4 (0.7) |
|  |  |  |
| Functional Health: Mean (SD) |  | 81.4 (20.1) |
|  |  |  |
| Depressive Symptoms: Mean (SD) |  | 6.1 (5.2) |
|  |  |  |
| Loneliness: Mean (SD) |  | 1.8 (0.5) |
|  |  |  |
| Perceived Social Isolation: Mean (SD) |  | 1.6 (0.6) |

Sex and education were omitted from fixed effects regressions because they are time-constant. Thus, they remain constant throughout time for each individual.

Supplementary Table 2. Baseline characteristics of individuals ceasing to volunteer.

| Variables |  | Mean (SD)/n (%) |
| --- | --- | --- |
| Sex: N (%) |  |  |
| Men |  | 172 (56.0%) |
| Women |  | 135 (44.0%) |
|  |  |  |
| Age: Mean (SD) |  | 73.2 (5.4) |
|  |  |  |
| Marital Status: N (%) |  |  |
| Married, living together with spouse |  | 215 (70.3%) |
| Married, living separated from spouse |  | 4 (1.3%) |
| Divorced |  | 20 (6.5%) |
| Widowed |  | 53 (17.3%) |
| Single |  | 14 (4.6%) |
|  |  |  |
| Education Level: N (%) |  |  |
| Low (ISCED 0-2) |  | 21 (6.8%) |
| Medium (ISCED 3-4) |  | 152 (49.5%) |
| High (ISCED 5-6) |  | 134 (43.6%) |
|  |  |  |
| Employment Status: N (%) |  |  |
| Working |  | 3 (1.0%) |
| Retired |  | 293 (95.4%) |
| Other: not employed |  | 11 (3.6%) |
| Monthly Household Net Income: Mean (SD) |  | 2546 (1215) |
|  |  |  |
| Smoking: N (%) |  |  |
| I smoke daily |  | 18 (6.6%) |
| I smoke occasionally |  | 5 (1.8%) |
| I used to smoke, nut not anymore |  | 113 (41.4%) |
| I have never smoked |  | 137 (50.2%) |
|  |  |  |
| Alcohol Intake: N (%) |  |  |
| Daily |  | 40 (14.7%) |
| Several times a week |  | 71 (26.0%) |
| Once a week |  | 42 (15.4%) |
| 1 - 3 times per month |  | 29 (10.6%) |
| Less often |  | 65 (23.8%) |
| Never |  | 26 (9.5%) |
|  |  |  |
| Number of Chronic Conditions: Mean (SD) |  | 3.0 (1.8) |
|  |  |  |
| Self-rated Health: Mean (SD) |  | 2.4 (0.7) |
|  |  |  |
| Functional Health: Mean (SD) |  | 80.9 (22.0) |
|  |  |  |
| Depressive Symptoms: Mean (SD) |  | 5.5 (5.1) |
|  |  |  |
| Loneliness: Mean (SD) |  | 1.7 (0.5) |
|  |  |  |
| Perceived Social Isolation: Mean (SD) |  | 1.5 (0.5) |

Sex and education were omitted from fixed effects regressions because they are time-constant. Thus, they remain constant throughout time for each individual.
